# Communicative Methodology: Contributions to Social Impact Assessment in Psychological Research

**DOI:** 10.3389/fpsyg.2020.00286

**Published:** 2020-03-03

**Authors:** Gisela Redondo-Sama, Javier Díez-Palomar, Roger Campdepadrós, Teresa Morlà-Folch

**Affiliations:** ^1^Department of Psychology and Sociology, University of Zaragoza, Zaragoza, Spain; ^2^Department of Linguistic and Literary Education, and Teaching and Learning of Experimental Sciences and Mathematics University of Barcelona, Barcelona, Spain; ^3^Department of Business Studies, University of Girona, Girona, Spain; ^4^Department of Business Management, Universitat Rovira i Virgili, Tarragona, Spain

**Keywords:** impact assessment, communicative methodology, psychological research, social impact, methods

## Abstract

Recent advancements in the social impact assessment of science have shown the diverse methodologies being developed to monitor and evaluate the improvements for society as a result of research. These assessment methods include indicators to gather both quantitative and qualitative evidence of the social impact of science achieved in the short, medium, and long terms. In psychology, the impact of research has been mainly analyzed in relation to scientific publications in journals, but less is known about the methods for the social impact assessment of psychological research. Impact assessment in the domains of educational psychology and organizational psychology presents synergies with bottom-up approaches that include the voices of citizens and stakeholders in their analyses. Along these lines, the communicative methodology (CM) emerges as a methodology useful for the communicative evaluation of the social impact of research. Although the CM has widely demonstrated social impact in the social sciences, less is known about how it has been used and the impact achieved in psychological research. This article unpacks how to achieve social impact in psychology through the CM. In particular, it focuses on the theoretical underpinnings of the CM, the postulates linked to psychological research and some key actions for the implementation of the CM in relation to the design of Advisory Committees, working groups, and plenary meetings in research. Furthermore, it shows how the CM has been implemented in illustrative cases in psychological research. The article finishes with a conclusion and recommendations to further explore the ways in which the CM enables the social impact of research in psychology.

## Introduction

The social impact assessment of science is becoming crucial in the debates over research evaluation, influencing the way in which scientists conceptualize and develop their studies (Reale et al., 2017). The growing concern among researchers, funding agencies, universities, policy makers, stakeholders, and the general public regarding how science can result in concrete improvements for society, contributes to establishing research impact agendas in all scientific disciplines. The field of psychology has not been indifferent to this newly international trend. The Strategic Plan of the American Psychological Association, adopted in February 2019, has the mission “To promote the advancement, communication, and application of psychological science and knowledge to benefit society and improve lives” (APA, n.d., p. 5). In this context, there is a need to advance knowledge about the research methodologies that enable social impacts and the concrete ways to develop them.

The communicative methodology (CM) has been demonstrated to achieve social impacts in different fields of knowledge since it was conceptualized with the aim of being useful to society, contributing to improving individuals and collectivities under study and the society as a whole (Gómez et al., 2011). The CM addresses social demands for dialogue in research, including reflections and the providing of critical views of the social contexts (Gómez et al., 2006). The CM has the recognition of the European Commission (European Commission, 2010; Flecha and Soler, 2014) because of relevant research in the framework programs based on this methodology. It is important to consider that, in science, power claims and research dynamics can cause researchers to prioritize their status or benefits over improving people’s lives even if scientists are concerned with the improvement of lives. This approach can lead to cultural, gender, age or class biases and to exclusionary science and output. The CM contributes to transforming this concern in science, engaging the subjects in an intersubjective dialogue with researchers by means of which (with such engagement) it is possible “to develop new knowledge that can transform local conditions, as they shift from diagnosing social exclusion to identifying the approaches that work best to reduce it” (Flecha, 2014).

The improvement of lives and societies underscores the definition of social impact, which differs regarding the concepts of scientific and political impact. In an accurate review of the literature on the evaluation of impacts of research in the social sciences and humanities, Reale et al. (2017) related scientific impact to the capacity to found new schools of thought and influence future research, and they related political impact to the use of scientific knowledge by decision makers and/or social actors as the basis for policies and/or action (p. 300). Impact is also connected to broader societal goals, aiming at the improvement of the living conditions of individuals. Flecha defined social impact as the improvement of society and citizens in relation to their own goals, democratically settled as in the United Nations Sustainable Development Goals, for instance (European Commission, 2018).

On the basis of an extensive literature review and lessons learned from practices worldwide, Flecha articulated a set of quantitative and qualitative indicators, data sources and methodologies to measure social impact achievement in the short, medium, and long terms, drawing on the following key impact pathways: achieving R&I missions, addressing global challenges and engaging EU citizens. Flecha (2000) claimed that “social impact measurement will benefit from databases and repositories that collect evidence of social impact, which will play a similar role as the databases of scientific impact” (p. 56). Repositories and databases displaying evidence of social impact have emerged in recent years, including the Social Impact Open Repository (SIOR), the first one worldwide to store evidence of social impacts in all scientific fields on a free basis (Flecha et al., 2015). That SIOR is currently linked to Wikipedia and ORCID, two major international databases incorporating (and disseminating) scientific knowledge, is an indication of the growing importance that social impact has worldwide. SIOR (with Wikipedia and ORCID) includes a set of indicators to calculate the social impact of research projects. The definition of indicators is related to advancements in the development of methodologies to measure the impact of research and the assessment of the relevance of research priorities and topics for citizenship (European Commission, 2017).

The methodologies addressed to measure the impact of research activity have increased worldwide, but evaluation prevails in terms of scientific impact (Ravenscroft et al., 2017). However, the methods for assessing social impact are a major concern across scientific societies, funding research agencies, universities, etc. Most of the efforts to advance knowledge in this field, particularly in the social sciences and humanities, can be found in Europe (e.g., Framework Programme of the European Commission, Research Excellence Framework in the United Kingdom), North America (e.g., National Science Foundation), and the BRICS countries (e.g., Financer of Studies and Projects in Brazil, Department of Science and Technology in India). In the European context, it is important to emphasize the contributions led by the European Commission through the appointment in 2016 of the Expert Group on evaluation methodologies for the interim and ex-post evaluations of Horizon 2020, chaired by Flecha, and the subsequent publication of the report entitled “Applying relevance-assessing methodologies to Horizon 2020” (European Commission, 2017), which developed four methodologies to assess the relevance of European funding in framework programs: expert exploratory approaches using computer-based content analysis; expert exploratory approaches using human content analysis; text mining approaches, and social media approaches (top-down and bottom-up). This approach to measuring the social impact of research relies on the involvement of citizenship within the process of creating the criteria to define social impact. Flecha and his team (European Commission, 2017) drew on communicative methodology to create inclusive dialogic spaces for discussion, which is a remarkable contribution to the field of research assessment in the social sciences and humanities (including psychology). The guiding questions for the development of the four aforementioned methodologies were based on the institutional perspective, the citizen’s perspective, and the scientific and technological perspectives. With regard to the citizen’s perspective the question underlying the analysis was whether Horizon 2020 was in line with the needs of EU citizens.

The citizen’s perspective sets peoples’ needs and voices at the core of the dialogue between science and society. In psychology, similar to other fields in the social sciences and humanities, we can find similar trends in engaging target populations within the process of research [design, interpretation of the results, and/or validation and reliability (Radstake et al., 2009; Davies et al., 2008)]. These studies look forward advancing toward a more responsible interaction of psychology with society, including dialogue with vulnerable populations, for instance, indigenous people (Davidson et al., 2000), or patients with acute decompensation of psychiatric pathology (Moreno-Poyato et al., 2019). Furthermore, Bromme and Goldman (2014) explored the public understanding of science, analyzing the way in which people make decisions linked to psychology without a deep comprehension of research. In a similar vein, the research program *Science with and for Society* (Swafs) of the European Commission includes projects attempting to bridge the gap between the scientific community and society at large, with the presence of psychological research in case studies selected in the Ex-post Evaluation of Science in Society in FP7 (European Commission, 2015).

This international trend of including people’s voices (demands, needs, etc.) within the process of research assessment (and the design of new research framework programs) tends to be built from top-down approaches and therefore from the researcher’s point of view, instead of citizens’ views (bottom-up) (Rau et al., 2018). Complementary to the use of top-down approaches, the methodologies linked to bottom-up could contribute to articulating a comprehensive understanding of the social impact of research in psychology. For instance, the use of methodologies to assess social impact in social media capture citizens’ opinions about the improvement of daily lives after the implementation of research (Cabré-Olivé et al., 2017; Pulido et al., 2018). In the light of this relationship, the CM becomes very useful as a methodological approach that include people’s voices from a bottom-up approach (Gómez, 2015).

In this article, we discuss how using the communicative methodological approach to research conducted in the field of psychology could reach remarkable levels of social impact. We aim to unpack how to achieve a social impact in psychological research through the CM. Communicative impact assessment of the research is used as a method to discuss the aforementioned goal. We first present the advancements in the social impact assessment of psychological research, including the methodologies used to evaluate programs, research projects, and evidence-based interventions in psychology research that have achieved impact. Then, we explain the theoretical underpinnings of the CM and the postulates linked to psychological research to clarify how can we use the CM approach as a methodological instrument to conduct the discussion. Then, we analyze communicative research in psychology in order to address the aims stated above. Finally, we present the article’s conclusion and limitations to further explore the methodologies linked to the social impact of research in psychology.

## The Communicative Methodology As A Methodological Approach To Assess Social Impact In Psychological Research

The CM is widely recognized as a useful methodology to achieve social impact through research since it allows for study not only of the exclusionary elements that reproduce inequalities but also of those elements contributing to overcome them (European Commission, 2011; Gómez et al., 2011; Gómez, 2017; Díez-Palomar et al., 2018). It is implemented in diverse disciplines in the social sciences, including sociology (Flecha and Soler, 2014), gender studies (Puigvert, 2014), and physical education (Castanedo and Capllonch, 2018), among others. This methodology “implies a continuous and egalitarian dialogue among researchers and the people involved in the communities and realities being studied” (Gómez et al., 2011). The role of the researchers is to bring scientific knowledge to the discussion, while the subjects contribute with their knowledge from their *lifeworlds* [in Schütz’s (1967) terms]. On the basis of this dialogical process, it is common for new understandings of social realities to flourish, informing potential answers to social problems. The dialogue and inclusion of people’s voices throughout the research process create transformative synergies in the field of psychology, as reported by Racionero and Padrós (2010).

The CM draws on the ontological assumption that “reality” is somehow “communicative.” That is, it is a human construction in which the meanings associated with “things” are built in a communicative manner through the interactions between individuals. In epistemological terms, the CM is dialogical in nature since the scientific statements employed in the discussion of the evidence are the result of a dialogue based on the intersubjectivity (Stolorow et al., 1994; Gómez et al., 2006) of the participants in the research. The social orientation of the CM is to transform the social context through communicative action (Habermas, 1984; Soler and Flecha, 2010), applying quantitative and qualitative techniques. The CM draws on seven postulates (Gómez et al., 2006, 2019):

– Universality of language and action;
– Individuals as transformative social agents;
– Communicative rationality;
– Common sense;
– Disappearance of the premise of an interpretative hierarchy;
– Equal epistemological levels; and
– Dialogic knowledge.

In this article, we use illustrative cases in psychological research to discuss how they have used communicative research methods to assess social impact. Table 1 summarizes the underlying postulates to discuss the advancements in the impact assessment methods among the illustrative cases chosen. As far as we know, the CM in psychological research was used in these cases. Codes are aligned with the seven postulates of the CM, as explained above.

**TABLE 1:** Coding scheme drawing on the seven postulates of the communicative methodology.

**Postulate**	**Definition**	**Code**	**Use in psychological research**
Universality of language and action	Language and action are inherent capacities of all human beings (Habermas, 1984; Chomsky, 1996). There is no hierarchy between cultures, ages or genders to develop cognitive and communicative capacities that allow them to interpret the world.	ULA	This postulate implies that professionals in psychology, patients, therapists, counselors, caregivers, families of patients, patients’ associations, and other members linked to psychology have the capacity to interact with others to express their views, including the evaluation of an intervention or program.
Individuals as transformative social agents	Individuals have the capacity to interpret the world and undertake actions addressed to its transformation and change.	ITA	Vygotsky (1978) argued that language is the symbolic tool that aids cognitive development, allowing individuals to interact toward change. In this vein, Bruner (2012) posited that transformation addresses human nature, instead of biological adaptation.
Communicative rationality	According to Habermas (1984): “the concept of communicative rationality has to be analyzed in connection with achieving understanding in language. The concept of reaching an understanding suggests a rationally motivated agreement among participants that is measured against criticizable validity claims” (p. 75).	CR	The postulate of communicative rationality in psychology suggests that researchers or other members enter into a scaffolding dialogue to improve the assessment processes and methods. The ultimate aim is to benefit the whole impact evaluation community.
Common sense	Individuals acquire diversity of knowledge and beliefs that influence their comprehension of the world and common sense (Schütz, 1967). This background influences the interpretation of reality, and the cultural contexts provide meaning to thoughts and actions (Rogoff, 2003).	CS	The link between the CM and social impact evaluation on the basis of the postulate of common sense includes open channels of dialogue and interactions that embrace different views and background knowledge of very diverse agents, from practitioners to researchers or patients.
Disappearance of the premise of an interpretative hierarchy	Beck addresses how the desmonopolization of experts’ knowledge occurs in the context of a risk society, paying special attention to the role of reflexivity (Beck et al., 1994). In the analysis by Lash of Beck’s conception of reflexivity, the author states that “reflexivity and modernity entail a growing freedom from and critique of expert-systems. Structural reflexivity thus involves freedom from the expert-systems of dominant science. Self-reflexivity involves a freedom from and critique of various psychotherapies. Reflexivity is based not in trust but in distrust of expert-systems” (Beck et al., 1994, p. 116).	DIH	The interpretations of academic and non-academic audiences have the same value. Therefore, in the evaluation of social impact framed by the CM, the best arguments from users or scientists can improve the assessment processes.
Equal epistemological level	Participants and researchers are at an equal epistemological level to understand the social reality and participate in a research process. The contributions that researchers and non-academic make to research are different since the knowledge that they have is also diverse. The knowledge coming from the individuals is experience and daily life learning, while researchers provide scientific knowledge.	EEL	The equal epistemological level of the CM implies a more precise analysis and understanding of psychological and social problems. In the field of social impact assessment in relation to this postulate, the evaluative arguments from non-academic audiences are equally valid and useful for developing and improving them.
Dialogic knowledge	The CM of research includes the objectivity and subjectivity perspectives to advance toward a dual perspective of the world that recognizes at the same level the structures (systems) and the life world. The intersubjective perspective underlines the interpretation of reality and generation of new knowledge, which are influenced by the people’s environments and meanings of reality (Flecha, 2000; Mercer, 2000). The construction of evaluation knowledge is grounded in dialogue since individuals accumulate knowledge using dialogue (Howe and Abedin, 2013).	DK	Impact assessment methods linked to the CM can achieve more accurate results in the evaluation processes since dialogue includes diverse views, reflections, voices, needs, and perspectives from different agents.

## Toward the Implementation of the Communicative Methodology With Regard to Social Impact

The CM includes in the research organization key procedures for the design, implementation, and analysis of data. The communicative organization of research is a concrete methodological dimension that allows for social impact assessment on short-, medium-, and long-term bases. There are three actions related to the communicative organization of research that are particularly relevant in terms of social impact assessment: the creation of the Advisory Committee; the definition of working groups; and the planning of plenary meetings. These actions are foreseen since the beginning of a research project, and they play the common role of following up the social impact of research results and guiding potential corrections during the research process. It is important to emphasize that egalitarian dialogue underpins the three actions and works as a cross-feature. The research team has the responsibility of ensuring that the functioning procedures and protocols of the Advisory Committee, working groups, and plenary meetings are transparent. Furthermore, there are mechanisms to ensure that they can be improved during the research project, taking advantage of the contributions of the users’ views. Diversity is crucial to developing these actions successfully.

### Designing the Creation of the Advisory Committee

The Advisory Committee of a research project implies the creation of a group of individuals who represent the communities studied. For instance, if a study focuses on the psychological effects of consuming alcohol during adolescence, the Advisory Committee should include young people with this problem to better approach the reality and to attain understanding and potential solutions. These committees usually have two representatives of the study group who interact with researchers on the basis of an equalitarian dialogue, accomplishing the postulates of the CM. The representatives bring their knowledge to the research process, and they can review the research guides and reports, questionnaires for the fieldwork, and other materials. It is important to emphasize that they actively contribute to transforming and improving the initial situation of the vulnerable group. The role of the Advisory Committee in the evaluation process is crucial to achieving a social impact since it is composed of representatives of the study groups, and it can play a role in *ex ante*, *in itinere*, or *ex post* stages of research. The methodologies for collecting their views about social impact evaluation can be quantitative and qualitative.

### Defining Working Groups

Science requires a research background from several fields to advance knowledge. Interdisciplinarity has grown worldwide, and it is common to collaborate between disciplines at the international, national, or regional level. In the case of the CM, research can include operational subgroups o focus on particular topics or tasks. For example, in a research project approaching the psychological impact of the use of technologies in adulthood, the working groups could include the fields of psychology, communication, sociology, and/or adult education. Each of the subgroups works fluently and can have diverse responsibilities during the research process. Volunteers who are experts in specific domains can participate in them. The Advisory Committee and coordination research team discuss the advancements and/or proposals of the working groups. The role of the working groups in the social impact assessment is mainly *in itinere* since they are operative mainly during the research process. The communication flow with the Advisory Committee is one of the most important aspects for reviewing and mitigating potential problems that can reduce social impact of research. As in the case of the Advisory Committee, the methods to capture insights regarding social impact evaluation can be quantitative and qualitative.

### Planning Plenary Meetings

The plenary meetings include all research members in a forum that can be addressed in ways to achieve social impact, evaluate the utility of research methods or design dissemination strategies, among other issues. The Advisory Committee receives the results of the plenary meetings to assess them and evaluate whether they require further improvements. Furthermore, at the end of the project the research team organizes a final conference addressed to stakeholders and end users. The aim is to engage all of the agents in a dialogue that includes the social impact assessment of the research results. In the case of psychological research, the final conference can have patients as speakers, presenting the benefits of a study together with researchers. Sometimes, these speakers are members of the Advisory Committee, and as occurs in the case of the working groups, the flow between the members of the plenary meetings and the Advisory Committee is a very relevant dimension of the communicative organization of research. The timing, planning, and flexibility of the organization of the plenary meetings depend on the identification of emerging needs during the research process.

## Illustrative Cases of the CM Used in Psychological Research Achieving Social Impact

There are three moments at which social impact assessment occurs (*ex ante*, *in itinere*, and *ex post*), and the actions of the CM can play different roles in each of them. *Ex ante* evaluation of social impact is when potential (not real) social impact is evaluated, and it is the most challenging one. During the implementation of research, that is, *in itinere*, it is possible to identify possible mistakes with regard to social impact and to mitigate them. Once the research finishes, social impact evaluation can also be undertaken on an *ex post* basis. In this section, we illustrate through two selected cases (Denzin and Lincoln, 2011) how CM reaches remarkable levels of social impact in psychological research. These cases provide relevant details to inspire other researchers develop studies applying MC to achieve social impact in psychological research.

### Illustrative Case 1: The CHIPE Project

The EU-funded research project “Children’s Personal Epistemologies: Capitalizing Children’s and Families’ Knowledge in Schools Towards Effective Teaching and Learning” (García-Carrión, 2013-2015) provided relevant results for improving children’s cognitive and social development, and the use of the CM was particularly important to evaluating the social impact *in itinere*. The project part of actions help citizens to succeed in education for subsequent access to the labor market and full participation in society. On the one hand, it focuses on a better understanding of the role of personal epistemology in schools that contributes to developing and consolidating innovative educational practices in education systems. On the other hand, the enhancement of effective teaching and learning in dialogic learning environments lays the foundation for providing people with more and better skills and competencies.

The researchers planned and designed the interventions in dialogue with the teachers, students, and family members from the beginning to the end of the project (codes *ULA* and *EEL*, Table 1). Furthermore, they were involved in regular meetings conducted at the university, as well as in their homes and communities. Sharing these dialogues enabled the researchers to “find ways of producing meaningful, accessible, and evocative research to understand people’s experience intersubjectively” (García-Carrión, 2015, p. 918). That illustrated the impact of CM drawing on the *DIH* postulate, in order to generate *dialogic knowledge* (Table 1). Ultimately, children engaged in higher order interactions (Hargreaves and García-Carrión, 2016) and developed solidarity-based relationships in classrooms where they felt valued and included (Villardón-Gallego et al., 2018). The implementation of the CM allowed for assessing the social impact during the whole research process, enabling improvements to expand it. This is also a remarkable example of how CM enables individuals to show their nature as transformative social agents (code *ITA*, Table 1).

The CM was used with different techniques, for instance, participant observations in the classrooms that evaluated successful educational performances, as well as interviews with students, families, teachers, and other community members. The use of those techniques are aligned with the CM assumptions summarized in Table 1 (in particular, *CR*, *CS*, *DIH*, and *DK*). The research team participated actively in classroom activities. At the end of each session, the researcher discussed the observations with the teacher and the volunteers – whenever it was possible – to also include their perspectives and impressions; that is, the participants played an active part in the entire research process. The results of their participation and of being involved in the transformation and educational impact that the execution of Successful Educational Actions had for the students are evidenced through the project, families, and volunteers becoming active members of the school and taking responsibility for the children’s education; for example, one of them played an active role as a governor as a direct result of participating in interactive groups after the research.

According to the European Commission summary publication of results, the “CHIPE outcomes included improved academic achievement, especially in economically deprived areas,” and “The project team concluded that a pupil-focused dialogic environment improves academic achievement, produces complex linguistic constructs and encourages students to draw upon their knowledge. Furthermore, the technique was seen to produce discussion about moral, taboo and/or difficult topics, and yielded positive social relationships.”

### Illustrative Case 2: MEMO4LOVE Project

The incorporation of participants’ voices throughout the process, which is characteristic of the CM, is innovative in psychological studies of memory since, in studies related to memory quality, it is common for participants to write their memories but exceptional that the participants themselves create a dialogue of their written memories. The written record of dialogue provides a finer interpretation of the interpretation itself. On the one hand, it allows the researcher to better understand the quality of memory, which means more details. On the other hand, the participant is more aware of the meaning and impact of the intervention in question.

The CM starts from the premise that participants in research are transforming agents since, through reflection, we produce our own practices, and we are able to intervene and transform social structures (Table 1). In this sense, MEMO4LOVE involved families, teachers, and students from the beginning of the research, providing contact with researchers through informative sessions, at which they could ask questions of the researchers. Similarly, following the CM, an advisory council was created with expert researchers who were not part of the research team, distinguished people at the international level of NGOs, and people from the administration and education fields, all of whom had participated since the beginning of the investigation.

In the project, questionnaires were designed that were validated by the adolescents themselves. That is, the principle of equal dialogue is present in all phases of the project. The project details that the relevance of the inclusion of the voices had two causes: first, because this interpretive approach is scarce in the area when assessing the impact of prevention programs; and second, because existing scales measuring gender-based violence victimization and gender violence attitudes do not contemplate the most common types of first sexual-affective relationships among adolescents, which are sporadic, and in which much violence occurs (Puigvert et al., 2019).

The project has been consolidated with the participants, results have been jointly developed, and proposals for interventions have been modeled. Similarly, the issue of consent in sex-affective relationships was incorporated into the project – a topic that was in the public debate – and due to the agents’ concerns, it was decided to add this concept to the study. That is, the principles of the CM in allowing the addressing of social problems and detailing the transforming factors caused the topics addressed in the research to have very much in mind the social context of the moment, and together with the participants, they adapted to the new realities, as the MEMO4LOVE project also did. From active participation in the research, agents knew the actions and could disseminate them in their contexts. That is, there was greater dialogue and greater interpretation, creating a greater sense and meaning of research in their lives.

One of the results of this line of research was the contribution entitled “Reconstruction of Autobiographical Memories of Violent Sexual-Affective Relationships through Scientific Reading on Love. A Psycho-Educational Intervention to Prevent Gender Violence” (Racionero-Plaza et al., 2018). With regard to the CM, it is explained that, before participants were involved in the study, the researchers informed them about the research, and they completed written informed consent forms. Research participants had time to read the consent form and to ask questions of the researchers. Explanations were provided by the researchers when necessary. The information provided on the consent form explained the objective of the study, the voluntary nature of participation, the possibility of withdrawing from the study at any time, the procedure to collect the data, the materials and measures to be used, and the anonymity and privacy statements. Since the article describes the methodological perspective of the research, it allows for adapting the focus to the needs of the participants, as well as bringing research closer to social reality.

The reading of the book generated such an impact among the participants that, once they read the book and engaged in dialogic relationships with the researchers, they asked to hold a meeting to deepen their understanding of the topic. Following the principle of the CM of responding to the social needs that arose, a focus group was conducted at the same time, and to adapt the research to the needs of the participants, the ethical codes of the research are very present. The same article reflects how the participants reflected on the relationship with or without violence, and as a result of this dialogue, a participant decided to end a current relationship.

## Conclusion

The aim of this article has been to provide an overview of the impact assessment methods in psychological research and the relevance that the CM has in this field.

The analysis demonstrates that, although scientific impact has played a key role in the impact assessment of psychological research, the concerns and contributions regarding how to measure social impact have increased over the years. To this end, the following points are crucial. First, it is important to consider the diversity of voices that can participate in impact evaluation processes, in an effort to advance toward the co-creation or co-production of psychological knowledge. Second, CM emerges as a useful methodology to contribute to social impact assessments in psychological research. Third, the underlying postulates and the concrete strategies of the CM create a research environment that facilitates the serving of society. The illustrative cases in psychological research provide evidence of the implementation of the CM in this field. Psychological research plays a crucial role in the improvement of societies, and the use of the CM has the potential to increase the social impact of psychology. In doing so, not only can the gap between science and society can be reduced, but it also is possible to open new horizons to achieve more and better psychological research that continues to improve people’s lives.

## Data Availability Statement

The datasets generated for this study are available on request to the corresponding author.

## Author Contributions

GR-S, JD-P, RC, and TM-F made substantial contributions to the conception of the work or the acquisition, analysis, or interpretation of the data for the work, drafted the work or revised it critically for important intellectual content, provided approval for publication of the content, and agreed to be accountable for all of the aspects of the work in ensuring that questions related to the accuracy or integrity of any part of the work are appropriately investigated and resolved.

## Conflict of Interest

The authors declare that the research was conducted in the absence of any commercial or financial relationships that could be construed as a potential conflict of interest.
